# Stakeholder views on the barriers and facilitators of psychosocial interventions to address reduction in aggressive challenging behaviour in adults with intellectual disabilities

**DOI:** 10.3310/nihropenres.13437.2

**Published:** 2023-12-29

**Authors:** Athanasia Kouroupa, Leila Hamza, Aisha Rafiq, Angela Hassiotis, Penny Rapaport, Andrew Jahoda, Laurence Taggart, Liz Steed, Sally-Ann Cooper, Craig Melville, Louise Marston, Rachel Royston, Afia Ali

**Affiliations:** 1 Division of Psychiatry, Faculty of Brain Sciences, University College London, London, England, UK; 2 Assessment and Intervention Team, Barnet Enfield and Haringey Mental Health NHS Trust, London, England, UK; 3 School of Health and Wellbeing, University of Glasgow, Glasgow, Scotland, UK; 4 Ulster University, Coleraine, Northern Ireland, UK; 5 Centre for Primary Care, Wolfson Institute of Population Health, Queen Mary University of London, London, England, UK; 6 Department of Primary Care and Population Health, Institute of Epidemiology and Health Care, University College London, London, England, UK; 7 Unit for Social and Community Psychiatry, Queen Mary University of London, London, England, UK

**Keywords:** Intellectual disabilities, aggression, challenging behaviour, qualitative methods, community care, interventions

## Abstract

Success of psychosocial interventions in reducing aggressive challenging behaviour is likely to be related not only to mechanistic aspects but also to therapeutic and system factors. The study aims to examine the facilitators and barriers that influence whether psychosocial interventions for aggressive challenging behaviour in adults with intellectual disabilities lead to positive change. We conducted 42 semi-structured interviews with adults with intellectual disabilities who display aggressive challenging behaviour, family/paid carers, and professionals engaged in or delivering a psychosocial intervention across the UK. Data were analysed thematically using a framework approach. Stakeholders considered therapeutic and supportive relationships and personalised care as facilitating factors in addressing aggressive challenging behaviour. The operational structure of community intellectual disability services and conflicting expectations of professionals and carers were the main contextual barriers that impeded the implementation of psychosocial interventions in adults with intellectual disabilities. Findings highlight the valued components that maximise positive change in adults with intellectual disabilities who display aggressive challenging behaviour. Several operational adjustments including referral criteria, roles of professionals and workforce issues need to be addressed in services to maximise the implementation of psychosocial interventions leading to reduction in aggressive challenging behaviour in this population.

## Introduction

Aggressive challenging behaviour is reported in 8% of adults with intellectual disabilities known to services ( Bowring *et al.*, 2017) with rates up to 53% for verbal aggression followed by 48% for physical aggression across settings ( *e.g.*, community group homes, residential settings) ( O’Dwyer *et al.*, 2018). Examples of aggressive challenging behaviour might include incidents of damage to property and/or antisocial behaviour ( Deb *et al.*, 2016). Aggressive challenging behaviour in adults with intellectual disabilities are often triggered by environmental factors including unexpected changes and/or demands to the person as well as other factors such as medication, mental health conditions, and level of intellectual functioning ( van den Bogaard *et al.*, 2018).

Existing interventions for aggressive challenging behaviour in adults with intellectual disabilities living in the community include behavioural approaches such as Positive Behavioural Support (PBS) and/or Applied Behavioural Analysis (ABA), Cognitive Behaviour Therapy (CBT) informed anger management along with pharmacological ( *e.g.*, antipsychotic medication) and alternative approaches ( *e.g.*, art therapy, sensory integration) ( NICE, 2015). Nonetheless, there is limited evidence for other psychosocial approaches which may be helpful to some adults with intellectual disabilities who display aggressive challenging behaviour such as Dialectical Behaviour Therapy (DBT) ( Brown *et al.*, 2013) and mindfulness ( Singh *et al.*, 2013; Singh *et al.*, 2016; Singh *et al.*, 2020). Psychosocial interventions aim to meet unmet needs of adults with intellectual disabilities including housing, self-care, social and interpersonal skills, daytime activities, and housekeeping for those in family, supported or residential care homes ( Gustafsson *et al.*, 2009).

Although, the evidence-base and benefits of psychosocial interventions for reducing aggressive challenging behaviour in adults with intellectual disabilities are undisputed ( Browne & Smith, 2018; Vereenooghe & Langdon, 2013), it is accepted that not all adults with intellectual disabilities respond to them. In addition, there is scarce understanding of how those who receive these interventions experience them and what their expectations are. The presence of aggressive challenging behaviour impacts not only the well-being of the individual with intellectual disability who might or might not be able to communicate their needs ( van den Bogaard *et al.*, 2018), but also has consequences for the surrounding environment including family members and/or paid carers who might be recipients of aggressive incidents ( van den Bogaard *et al.*, 2018).

Research has explored the mechanisms that maximise engagement with psychosocial therapy for adults with intellectual disabilities. A qualitative study described the facilitators and barriers of positive therapeutic change ( *i.e.*, engagement in therapy, generalisation of acquired skills) following access to mental health services ( Ramsden *et al.*, 2016). Triads of six adults with mild intellectual disability who had accessed and benefited from the service, their (six) carers and three clinical psychologists working in the service agreed that a supportive network, a good therapeutic relationship, and adaptations in sessions facilitated positive therapeutic change, whereas communication and cognitive limitations as well as acceptance of the problematic behaviour impeded engagement in therapy ( Ramsden *et al.*, 2016). Other studies explored the views of multiple stakeholders about PBS implementation in different settings ( *i.e.*, inpatient mental health, forensic, community intellectual disability services) ( Bosco *et al.*, 2019; Clark *et al.*, 2020; Karger *et al.*, 2018). Professionals, carers (family or paid) and adults with mild intellectual disabilities described PBS as a beneficial approach to get to know the needs of individuals which in turn may promote holistic care ( Bosco *et al.*, 2019; Clark *et al.*, 2020; Karger *et al.*, 2018). However, professionals and care home managers emphasised that PBS plans were not only poorly implemented, but there was confusion about their purpose and use within the team ( Clark *et al.*, 2020). Barriers to PBS implementation included differences in staff attitudes towards and knowledge of PBS, resistance to change, organisational issues such as understaffing, and poor motivation and engagement of staff due to PBS being perceived as time consuming ( Bosco *et al.*, 2019; Clark *et al.*, 2020; Karger *et al.*, 2018). Overall, they all perceived PBS as leading to an improved collaborative and personalised approach to patient care and, therefore, were willing to adopt it in their effort to reduce restrictive practices ( Bosco *et al.*, 2019; Clark *et al.*, 2020).

A recent review summarised the perceived facilitators and barriers to the use of Active Support in adults with intellectual disabilities in residential care ( Flynn *et al.*, 2018). Active Support can be described as a preventative intervention that aims to maximise opportunities for adults with intellectual disabilities to be active members in the community they live in, with supervision from care home staff members. However, evidence is inconclusive in relation to its effect on minimising challenging behaviours including aggression towards people and/or property among other behaviours ( Stancliffe *et al.*, 2008; Totsika *et al.*, 2008). The review findings suggested that training residential care staff, supervision, and peer support facilitated the implementation of Active Support in residential care for adults with intellectual disabilities ( Flynn *et al.*, 2018). Whereas, operational issues including staff retention, lack of support and leadership followed by low levels of staff motivation and absence of knowledge on how to manage challenging behaviours impeded the implementation of Active Support uptake by staff working in residential care ( Flynn *et al.*, 2018). Interestingly, a meta-analytic review suggested that following staff training, the attitude of care home staff working with adults with intellectual disabilities who display aggressive challenging behaviour changed but staff training itself was not effective in reducing aggressive challenging behaviour in adults with intellectual disabilities ( Knotter *et al.*, 2018).

Overall, there is limited in depth understanding of the experience of care and the reasons that promote or impede the reduction of aggressive challenging behaviour in adults with intellectual disabilities. This qualitative study explored the experiences of triads of adults with mild/moderate intellectual disabilities with a history of aggressive challenging behaviour, their family and/or paid carers, and health and social care professionals involved in their care of previously received psychosocial interventions. The objective was to better understand what constitutes facilitators and barriers in achieving positive change defined as reduction in aggressive challenging behaviour.

## Methods

### Patient and Public Involvement

The study recruited two panels of experts by experience, one with adults with intellectual disabilities (n=4) and the other with family carers (n=5) to shape the methodology. Participants information sheets, consent forms and topic guides for each stakeholder group were developed with input from researchers and the two panels of experts by experience. The two panels guided the analytic approach and participated actively in the interpretation of qualitative findings.

### Participants

In total, 14 triads (40 participants in total) were recruited comprising of an adult with mild/moderate intellectual disability with a history of aggressive challenging behaviour who had received treatment for their behaviours, their carer (paid and/or family), and a health or social care professional involved in their care. We interviewed triads of participants to explore in more depth and from multiple perspectives the convergence and divergence of opinions about the reduction in aggressive challenging behaviour in adults with intellectual disabilities following delivery of a psychosocial intervention. Participants were recruited via social care professionals from seven NHS community intellectual disability services across the UK ( *i.e.*, England, Scotland, and Northern Ireland) covering both urban and rural areas. Social care professionals received verbal assent from adults with mild/moderate intellectual disability with a history of aggressive challenging behaviour who had received treatment for their behaviours and their carer (paid and/or family) to be contacted by a researcher to receive detailed information about the study over email, phone or videoconference due to COVID-19 lockdown restrictions. Purposive sampling aimed to achieve participant variation in age, work experience, gender, professional background, and carer roles.

Adults with intellectual disabilities were included if they: 1) had mild or moderate intellectual disability based on service records from the community team; 2) were aged 18 years or over; 3) had received a psychosocial intervention known to reduce challenging behaviour where aggression was the main feature such as verbal of physical aggression to people and/or property; 4) were able to speak English; and 5) provided consent to take part in the study. Carers were included if they: 1) were either a paid or family carer who was well informed of the person’s care plan; 2) were aged over 18 years; 3) able to speak English; and 4) provided consent to take part in the study. Health and social care professionals were included if they: 1) were aged 18 years or over; 2) had been involved in the care of the person with intellectual disability; and 3) provided consent to take part in the study. Exclusion criteria for adults with intellectual disabilities included: 1) severe intellectual disability; 2) insufficient verbal ability to take part in the interview; 3) lack of mental capacity to consent. There were no further exclusion criteria for carers and health and social care professionals.

### Ethics statement

Recruitment took place between November 2020 and May 2021 which included the period of the second COVID-19 lockdown in the three UK countries (England, Scotland, and Northern Ireland). Verbal audio-recorded informed consent was obtained from all participants which was then transferred to a paper form, as due to COVID-19 lockdown restrictions, we conducted individual interviews with each stakeholder member remotely ( *e.g.*, Zoom, Microsoft Teams, telephone calls). The study received NHS Health Research Authority approval from the East of England – Essex Research Ethics Committee (20/EE/0211).

### Procedure

Topic guides covered a broad range of topics including views about the nature of the participant’s behaviour, type, and experience of support they had received, positive and negative aspects of care and intervention received ( Kouroupa *et al.*, 2023). Adults with intellectual disability gave the research team permission to contact their carer ( *e.g.*, family and/or paid) and a health and social care professional involved in their care from the local community intellectual disability team ( *e.g.*, psychologists, psychiatrist, nurses). Researchers were experienced users of the online communication platforms ( *i.e.*, Zoom, Microsoft Teams). The mean duration of interviews was 45 minutes ranging from 18–97 minutes.

### Data coding and analysis

Interviews were audio-recorded and transcribed verbatim by an external sponsor approved agency. All identifiable information was removed. Data were analysed using the framework method for thematic analysis of interview transcripts ( Braun & Clarke, 2006; Gale *et al.*, 2013; Ritchie *et al.*, 2013). The framework method enabled the open, critical, and reflexive comparison and contrast of data from each triadic group to be compared across themes ( Gale *et al.*, 2013). The framework analysis provides a systematic and flexible approach to managing data from different stakeholders and allows for several members of the research team to contribute to data analysis ( Gale *et al.*, 2013). It also permits the use of both inductive and deductive approaches ( Gale *et al.*, 2013). Data from each participant group was initially analysed separately using NVivo software (version 11). Other qualitative data analysis software with similar functionalities may be used. Following review of the emerging themes and convergence of opinions from all stakeholder groups, themes are presented together. The process included: 1) data familiarisation; 2) identifying themes; 3) indexing of themes; 4) charting and summarising data; 5) interpreting and mapping data; 6) compiling this report. Three researchers (LH, AA, and AR) independently familiarised themselves with five transcripts to develop the initial coding frame for each participant group which was used as a basis for analysis the remaining transcripts. However, as further transcripts were analysed, new codes were added, and the coding frame was continually revised throughout this process. Codes were refined into broader categories ( *e.g.*, themes) to systematically explore convergence and divergence across the entire dataset and to develop themes related to facilitators and barriers.

## Results

We interviewed 14 adults with intellectual disabilities aged 19–47 years (Mean=33 years old; SD=10). Adults with intellectual disabilities had other neurodevelopmental ( *e.g.*, autism, attention deficit hyperactivity disorder) and/or mental health conditions ( *e.g.*, schizophrenia, bipolar disorder, anxiety, depression, obsessive compulsive disorder). Just over half of the adults (n=8) were residing in supported living accommodation the others were residing in some form of supported living/residential accommodation. In total, 13 carers took part in interviews most of whom were female (n=11). The mean age of carers was 53 years old (SD=9; range: 41–64 years old). Finally, 13 professionals with a mean of 16 years of employment post-qualification at a community intellectual disability service participated in the study. One paid carer and one health and social care professional participated in two triads each. Details of each triad are presented in Table 1, Table 2 and Table 3.

**Table 1.** Socio-demographic characteristics of adults with intellectual disability.

Characteristics	Adult with intellectual disability (n=14)
Age in years	
Mean (SD), range	33 (10), 19–47
Gender	
Male	10
Female	4
Accommodation	
Family home	6
Supported living	8
Number of additional diagnoses	
Mean (SD), range per person	2.07 (0.75), 1–3
Mental health problems ( *e.g.,* anxiety, depression, bipolar, OCD ^†^)	13
Neurodevelopmental conditions (e.g., autism, ADHD ^¶^)	9

^†^OCD: Obsessive Compulsive Disorder; ^¶^ADHD: Attention Deficit Hyperactivity Disorder

**Table 2.** Socio-demographic characteristics of carers.

Characteristics	Carers (n=13)
Age in years	
Mean (SD), range	53 (9), 41 – 64
Gender	
Male	2
Female	11
Carer type	
Family carer	6
Paid carer	7

**Table 3.** Socio-demographic characteristics of professionals.

Characteristics	Professionals (n=13)
Gender	
Male	6
Female	7
Professional expertise	
Psychology	5
Psychiatry	2
Nursing	3
Social Work	3
Years of experience in intellectual disability	
Mean (SD), range	15.7 (15.0), 2–43

The most common psychosocial interventions reported by all stakeholders were Positive Behaviour Support (n=7), Cognitive Behaviour Therapy (n=5) and Dialectical Behaviour Therapy (n=2). Only one person had received all three psychosocial interventions.

**Figure 1.**
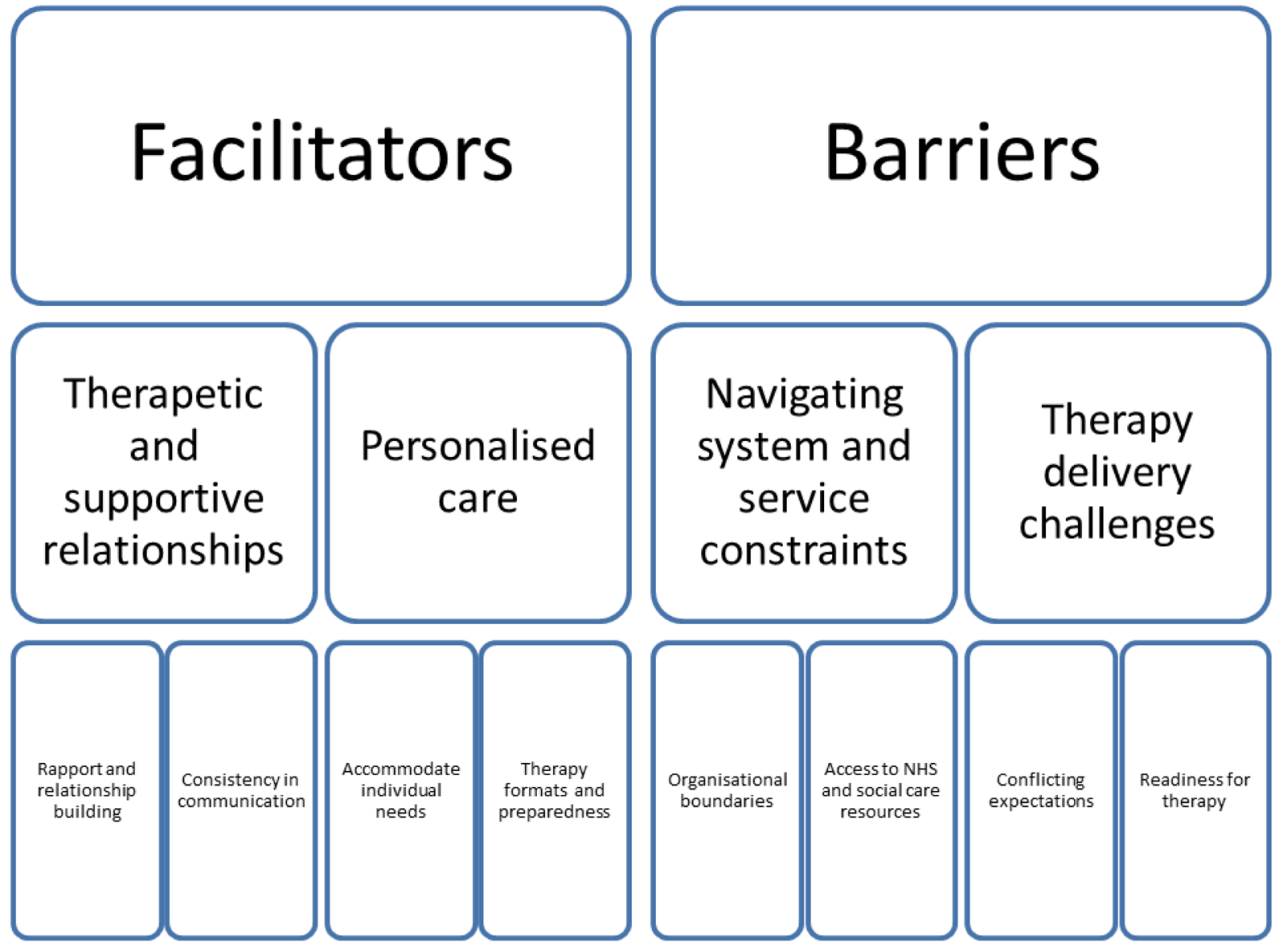
Summary of coding framework.

The coding framework was organised into two main domains relating to facilitators of and barriers to positive change ( *i.e.,* reduction in aggressive challenging behaviour) (see Figure 1).

### Facilitators of positive change following delivery of a psychosocial intervention for aggressive challenging behaviour

Therapeutic and supportive relationships and personalised care were the two main themes reported as having significant influence as to whether psychosocial interventions in adults with intellectual disabilities led to reduction in aggressive challenging behaviour ( Kouroupa *et al.*, 2023). All stakeholders shared common views on the important components that lead to positive change in adults with intellectual disabilities.

***Theme 1: Therapeutic and supportive relationships***

The term therapeutic and supportive relationships refer to the therapeutic alliance of carers ( *e.g.*, family and paid) and adults with intellectual disabilities with health and social care professionals and the broader supportive relationships of carers ( *e.g.*, family and paid) with adults with intellectual disabilities.

*Rapport and relationship building*

Developing a close and trusting relationship among all parties was important to all stakeholders. Professionals acknowledged that active listening to the person with intellectual disability and their carers ( *e.g.*, paid or family) facilitated better engagement in the intervention.

“The best treatment was the rapport…it took a few times to develop...If you press the right buttons…you need to have the confidence of the patient… Listening to them is very important. Initially he would not engage much, but once he built a rapport with us, then he was keen…more motivated to attend…It is a slow process.” (Consultant Psychiatrist)

People with intellectual disabilities and family carers emphasised the importance of the therapist’s positive interpersonal skills which motivated them to attend the sessions.

“They’re funny, they’re kind. They’re enthusiastic, they’re lovely. They help me to do things that I’ve never done before.” (Adult with intellectual disability)

Both adults with intellectual disabilities and family carers described how feeling genuinely listened to by professionals was important in developing trust and highly valued if they were to engage in an intervention.

“That she [therapist] understood what I was saying. She’d [therapist] give examples how to help and that...Willing to talk about it and not just shutting it off.” (Adult with intellectual disability)
“… they’ve all really taken onboard what the difficulties are for [1100011], and for us as well. I really genuinely feel that just being listened to has made a huge difference.” (Family carer)

Past negative experiences such as not being able to access services, care pauses or discontinuation and/or distrust of professionals appeared to influence current contacts with professionals as reported by both carers, particularly family carers, and adults with intellectual disabilities. Therefore, setting new expectations can also be beneficial in forming new positive contact.

“…I have managed to establish a positive relationship with mum… that has been really important because it would have been really easy to disengage from the family because mum, she doesn’t trust us. It would have been very easy for her, if we hadn’t been very careful in the way we had set things up around them, to disengage.” (Clinical Psychologist)
“I don’t tell anybody unless I trust them [therapists]…if it’s a new member of staff, I clam up…because I’ve been hurt that many times.” (Adult with intellectual disability)

Finally, the motivation of the adult with intellectual disability was a crucial enabling factor in promoting engagement in therapy.

“[800011]’s attitude to the therapy was brilliant. He really wanted to engage with it. There was a real desire to make changes…he was sort of a dream client really…as a credit to him he was so keen to come along and change.” (Assistant Psychologist)

*Consistency in communication*

Professionals focused on the role of carers ( *i.e.*, family or paid) as agents for intervention delivery and success.

“Having a small predictable support staff team was really vital…they had fewer people coming and going and he built a better relationship with them…they weren’t constantly revolving; they were a stable team which is really fantastic.” (Assistant Psychologist)

Professionals valued working with family carers and adults with intellectual disabilities to achieve a reduction in aggressive challenging behaviour and improve quality of life. Agreed care plans informed through discussions with both family carers and the person with intellectual disability led to a collaborative approach that increased confidence in the support network of a positive outcome.

“The best training possibly that we’ve had was actually family, was informal training for his mum and his sister. So, we linked in with the family, with the health professionals, with their own internal training. So, it was quite comprehensive across the board.” (Social Worker)
“She understood what I was saying. She’d give examples how to help and that...Willing to talk about it and not just shutting it off.” (Adult with intellectual disability)

In addition, professionals highlighted the value of accessing support from colleagues when dealing with complex cases as sustaining improvements and optimism.

“We’re all based in one building, we communicate any time we want about any matters, and we have regular meetings…we pass on information quickly and discuss matters, support each other, joint visits…It is a group effort, it’s not, I do one thing. We all know what each other is doing and we do seek advice from each other.” (Nurse)

Similarly, paid carers acknowledged the value of professional input when working with multiple individuals with complex needs.

“…We would have struggled without the support. Sometimes it just takes a fresh set of eyes coming in to see what we’re doing, reviewing the paperwork, and trying to make suggestions of what we could potentially try and do to improve it…” (Paid carer)

Family and paid carers focused on the importance of effective information sharing between professionals and carers to ensure a consistent approach is implemented across different contexts.

“… Everybody singing from the same hymn sheet. I have spoken to all the staff that works with [1100021] and I have made it very clear to them. You all need to be doing the same thing. It doesn’t work with [1100021] if somebody is doing one thing and somebody is doing another.” (Family carer)
“They are very involved, and then even in going to the meetings and that…it won’t be just, like, the same member of staff or the same team. It’ll be someone else will go so they understand exactly what goes into getting the care plan up and what the person actually needs…make sure that people understand why we’re doing things.” (Paid carer)

One family carer highlighted the importance of anticipating problems and ensuring that contingencies are built into treatment plans to help at times of transition. Such times can be unsettling and may lead to incidents of aggressive challenging behaviour in adults with intellectual disabilities.

“I would say the psychiatrist has been the most helpful…She seems very proactive in helping him and treating him. So, she’s putting in a…positive behaviour plan...She’s instrumental in getting that all organised for his move...I know that I can email or phone her if I’m worried about anything.” (Family carer)

Finally, professionals recognised the vital role of family and/or paid carers in supporting intervention attendance ( *e.g.*, organising and taking them to appointments) and practicing and implementing strategies in multiple contexts to maximise therapeutic gains.

“And having [carer] there consistently was really helpful. They had a really good work relationship, and she was able to continue that work outside and remind him of the stuff that we’d discussed. He’d always leave with homework…it was something that he practiced quite a lot, so he’d done really well to engage with that process. The support staff had been vital in supporting him to access that and reminding him that it would be helpful” (Assistant Psychologist)

***Theme 2: Personalised care***

Personalised care is defined as “supporting people with intellectual disabilities to build a lifestyle based on choices, preferences, shared power, rights and inclusion” ( Ratti *et al.*, 2016).

*Accommodate individual needs*

Professionals believed that focusing on the strengths and interests of the person with intellectual disabilities would facilitate engagement with the intervention.

“…my intervention has included now input from the Imam to work with him towards expressing himself, doing his prayers, but with reasonable adjustments…he would struggle with doing prayers five times a day, but he has this conflict that he’s not doing that. He’s very good with computers, he really engages well with computers, so the Imam…has sent him a digital Quran to work with …the support worker who’s also the same faith supports him in the prayers…” (Clinical Psychologist)

Adults with intellectual disabilities and family carers described the use of visual material or other aids ( *i.e.,* easy read booklets, ‘traffic light’ system) as helpful in understanding the behavioural strategies employed by professionals which in turn maintained the individual’s engagement.

“And the stickers to put on your door, I had all the stickers, red, green and amber...Red’s stop and green for go...Yes, green was always good...That can help me.” (Adult with intellectual disability)

Professionals reported that other factors such as humour may also improve engagement in a therapeutic conversation.

“…humour is a really good way to get [100021] engaged and positive, away from the negative… [100021]’s a big [football team] fan…he loves talking about that…. that’s a really good way of coaxing [100021].” (Social Worker)

Finally, adults with intellectual disability and professionals reported that the choice of words play a significant role in people’s engagement with services.

“Yes, I don’t like when some people say loads of words that can trigger me. But she [health and social care professional] was nice” (Adult with intellectual disability)
“He did mention…Psychology. I think he said, no and then with the behaviour support plan and all that, he sort of accepted, because the word Psychology is not there.” (Nurse)

*Therapy formats and preparedness*

The frequency and format of the intervention received were also seen as potential points for reasonable adjustments to maximise engagement in therapy.

“…there was a social element to the DBT…she was going along as part of a group. she enjoyed meeting new people, although it was difficult to begin with. Once she was established in the group, she enjoyed the company. She enjoyed the social element of that there. And then they could have a chat afterwards.” (Clinical Psychologist)
“Wouldn't do well in group therapy… he wouldn’t be able to concentrate, and also, he probably wouldn’t open us as much as he can do when it’s a one to one. Other staff members have to interpret it a little bit different if you’re in a group therapy you don’t upset your carer when you’re doing it in front of everybody, because otherwise he’d feel that you’re being put down in front of people and there’s all those massive impacts on them.” (Paid carer)

Family carers mentioned that it was helpful for them to be present during sessions to assist with communication and help to reassure the person.

“That’s why I think it’d be handy if I’m there as well. So, if I do find that it needs to be broken down, I can break it down. Because I’m used to how my kind of language with him works, than somebody else who’s mainstream doesn’t.” (Family carer)

### Barriers to positive change following delivery of a psychosocial intervention for aggressive challenging behaviour

Professionals, family carers and, less commonly, adults with intellectual disabilities focused on the problematic aspects at an operational level that impeded access to psychosocial support for aggressive challenging behaviour in adults with intellectual disabilities. The two main themes reported as a barrier were navigating through the system and service constraints and therapy delivery challenges.

***Theme 1: Navigating system and service constraints***

*Organisational boundaries*

Professionals raised the issue of eligibility criteria to access certain services ( *e.g.*, Improving Access to Psychological Services; IAPT) stemming from having or not having a confirmed diagnosis of intellectual disability.

“… We came across the IAPT* service…unfortunately because he has got a label attached to a learning disability, he was not accepted.” (Consultant Psychiatrist)
*IAPT: Improving Access to Psychological Therapy

In addition, there were issues with mainstream mental health services not being able to adapt interventions for adults with intellectual disabilities. Professionals within IAPT services were perceived as having preconceived ideas about psychological interventions like CBT not being a suitable approach for people with intellectual disabilities who display aggressive challenging behaviour.

“Yes, resources are always short, isn’t it? If he had a CBT therapist, people within IAPT, they don’t think he can do CBT…but I have done that model and I have found that he was going home, and he was maintaining the diary and he was coming back and talking about, there are alternative options.” (Consultant Psychiatrist)

Furthermore, professionals emphasised the difficulty in accessing clinic space to arrange sessions with adults with intellectual disabilities and/or carers.

“I wish we’d have had more access to facilities. I think part of the issue with the work that we did was with wanting to be consistent in terms of time and place. That can sometimes be a challenge. We don’t have… access to those clinic rooms…We shared them with the social services department…Having access to…an appropriate space would have been very helpful.” (Assistant Psychologist)

Finally, professionals and family carers reported lack of clarity around “whose job is this” to address aggressive challenging behaviour in adults with intellectual disabilities.

“…it’s just this really pervasive attitude…who’s taking responsibility for it? Is it psychology? Is it nursing that was picked up by nursing who just sent it back to me… I’m a psychiatrist. It’s usually not my job to provide ...” (Consultant Psychiatrist)
“…I don’t mean to sound like I’m against everybody, but I just honestly do believe so many people failed him.” (Family carer)

*Access to NHS and social care resources*

All stakeholders highlighted that service gaps ( *i.e.*, shortage of certain professions, *e.g.*, psychologists and nurses leading to long waiting lists for psychological support) in community intellectual disability services across different areas not only limit access to appropriate psychosocial interventions to address aggressive challenging behaviour in adults with intellectual disabilities but also likely to lead to potentially adverse consequences for the person with intellectual disability.

“This social worker…the things she’s promised like community transport, respite. She’s promised me to find out about clubs in the area, day centres...counselling…family therapy and all that lot? We’ve never heard of them.” (Family carer)
“I would love to…I’m still waiting…I just need that talking therapy. When’s it going to come? When it’s too late? …What do they want me to do?” (Adult with intellectual disability)
“Sadly, one of the things that I didn’t think we could offer, and it would have been…more helpful would have been family therapy. We used to have a family therapy service in [Town]. But that is not available now.” (Clinical Psychologist)

Similar issues apply to family carers who often need access to specialist support for themselves. However, such services are either unavailable or difficult to access due to other commitments.

“…no one’s suggested anything, or offered me anything…any support for myself, mental health” (100032 – Family carer)
“I am registered with the carers where they do offer things like mindfulness and massage and things like that which is super, but they are all during the day which I can’t go to because I work.” (Family carer)

In addition, inadequate paid carer support and high staff turnover in provider services is a fundamental problem in social care. As a result, repeated training of newly appointed staff is seen as additional burden which impacts the care of those individuals already referred for intervention.

“Staffing can be an ongoing issue really...Logistics and people calling in sick and new staff arriving that don’t know the care plans. That is an ongoing challenge.” (Nurse)

***Theme 2: Therapy delivery challenges***

*Conflicting expectations*

Professionals and family carers commented on the mismatch of what community intellectual disability services consider appropriate support to address aggressive challenging behaviour in adults with intellectual disabilities and service users’ expectations about access to support.

“… it’s not through lack of trying. I’ve just had the second referral for specialist psychology refused…he’s been consistent in saying he wants psychology input, and unfortunately, he’s not meeting the criteria for them to accept him because of his level of alcohol abuse and intoxication, they don’t believe that he’s in a place where he’s willing to buy into therapy and engage with it … There are some system failings…there are also some rigid criteria that he failed to meet which is inflexible.” (Nurse)
“They get the psychologist involved when things were really difficult…Unfortunately, it tended to be short bursts he would get…he would see a psychologist for maybe two or three months and then they would back off. Then when things got tough again, they would say oh, we will re-refer him. Then we had to go through the whole process all over again.” (Family carer)

Similarly, professionals emphasised that there are some carers who seek a ‘quick fix’ and become pessimistic when aggressive challenging behaviour persists often in the face of multiple cycles of an intervention.

“…she doesn’t consider that things have changed that much…I think her expectation is that she’s suddenly going to change completely.” (Clinical Psychologist)

*Readiness for therapy*

Professionals reported that it is often the case that carers find it challenging to implement the strategies suggested in the PBS plan consistently with adults with intellectual disabilities, therefore, aggressive challenging behaviours may be maintained. Regardless of the support and/or training provided, some individuals have additional needs which affect the care that is provided if not tailored to the specific individual needs.

“Although we tried to support the family to implement some of these strategies at home this has been very hard due to her father’s learning disabilities and her mother’s mental health issues. It’s difficult for them to process this information…his parents were not able to implement any recommendations that were being made to them. There are some also language barriers.” (100024 –Professional)
“They don’t help me. [Psychiatrist] might say, do this and do that. I forgot what she even said to me.” (Adult with intellectual disability)

Professional disagreements were also mentioned as potentially contributing to tensions in care planning, but such situations may not always be destructive if there is open communication between parties.

“There are disagreements all the time… you have to disagree to agree at some points and see where things are going.” (Paid carer)

Finally, professionals and paid carers highlighted that adults with intellectual disabilities may not always be ‘ready’ to engage in therapy due to a mental health condition.

“…but at times of crisis she really struggled with actually engaging in them...if you were to prompt…she’ll just look at you, but her eyes will be really wide…she can’t even process and there is a cognitive delay from one of the previous overdoses that she had taken…” (Paid carer)

## Discussion

This study illustrates the complexities of achieving positive outcomes following psychosocial interventions for aggressive challenging behaviour in adults with intellectual disabilities. Triads of stakeholders described the value of developing relationships and connecting with carers ( *e.g.*, family or paid) and/or adults with intellectual disabilities. All stakeholders valued the importance of developing care plans with carers ( *e.g.*, family or paid) and adults with intellectual disabilities, collaborating with other professionals, where needed, and monitoring during the implementation of care plans. Similarly, all stakeholders acknowledged the value of accommodating the needs of the person with intellectual disability in a session and making multiple adjustments to improve the experience of therapy. Nonetheless, all stakeholders identified problematic areas including access to and navigation through services and/or different views about the nature and limits of psychosocial interventions.

Our findings indicate that stakeholders across all NHS sites across the UK shared broadly similar views about the facilitators and barriers of addressing aggressive challenging behaviour in adults with intellectual disabilities. Some issues were more pertinent to people with intellectual disabilities and less so to carers or professionals and *vice versa*. Those inflection points are important gaps to be addressed but also underline the mismatch of expectations between stakeholders and services and the role of dynamic relationships among all parties involved while in psychosocial intervention to fully address the complexity of aggressive challenging behaviour. A recent study showed that the expected effect size of psychosocial interventions as reported in published clinical reports was much lower than the one expected by family carers and professionals ( Hassiotis *et al.*, 2022b). There is also evidence that behavioural interventions which are often first line approach for aggressive challenging behaviour may not be useful to all individuals with intellectual disabilities ( Woodcock & Blackwell, 2020).

Legislation and policy encourage professionals to collaborate with carers ( *e.g.*, family, paid) to complement and enhance their role in caring for vulnerable adults ( NHSE, 2015a; NHSE, 2015b) which was echoed in this study. Nonetheless, little is known about the dynamic relationship of those engaging in psychosocial interventions for aggressive challenging behaviour especially when family or paid carers are recipients of aggressive incidents. When viewed in the context of our results, this might explain why stakeholders described that the format of and preparedness to access psychosocial interventions was seen as a facilitator of positive change. A previous literature review described that psychosocial interventions were facilitated by individual factors (e.g., personalised care) and factors related to the immediate and/or wider social context of the person with intellectual disability with mental health conditions (e.g., supportive relationships with family or paid carers) ( Dagnan, 2007). Besides, our study aligns with the literature that carers value working with open-minded, sensitive, and skilled professionals to better understand aggressive challenging behaviour and developing care plans around the person’s needs, interests, communication abilities and preferences ( Botterill *et al.*, 2019; Cameron *et al.*, 2020; Deb *et al.*, 2022; Hassiotis *et al.*, 2022a; Surley & Dagnan, 2019; Tournier *et al.*, 2022). In line with this, adults with mild/moderate intellectual disability described that aggressive challenging behaviour reduces when they experience positive relationships ( Clarke *et al.*, 2019).

Deep-rooted systemic issues in community intellectual disability services that impede the implementation of psychosocial interventions for aggressive challenging behaviour included the organisational boundaries of a service ( *e.g.*, clarity of roles, referral criteria, no clinic space) and resource availability. There has been extensive literature addressing these ongoing problems in the NHS, including unclear roles within the same team, strict referral criteria to specialist services for adults with intellectual disabilities that can complicate the care pathway, high staff turnover and limited access to psychosocial therapies ( Brown *et al.*, 2016; Dagnan, 2007; Flynn *et al.*, 2018; Hassiotis *et al.*, 2022a; Marwood *et al.*, 2018). Research indicates that simplifying these procedures enhances engagement with professionals and therapy and facilitates behavioural change ( Botterill *et al.*, 2019).

There is scarce data to evaluate the implementation of psychosocial interventions for aggressive challenging behaviour in adults with intellectual disabilities to compare with this work ( Bambara *et al.*, 2001; Bradshaw *et al.*, 2004; Davies *et al.*, 2021; Lindsay *et al.*, 2003; Willner *et al.*, 2013). Pre-existing research focuses on the social care failure rather than exploring the implementation of psychosocial interventions to address aggressive challenging behaviour in adults with intellectual disabilities. Interestingly, a scoping review described that family carers were involved in the development and evaluation of care plans but not in implementation ( Tournier *et al.,* 2021). The input of family carers in care plans is undisputable. A consistent approach to implementing care plans across services followed by ongoing family involvement, is equally crucial to lead to a positive change. Future research should determine whether psychosocial interventions have been implemented as intended and result in the reduction of aggressive challenging behaviour in adults with intellectual disabilities in community and/or other settings. Future studies might consider interviewing carers (e.g., family and/or paid), adults with intellectual disabilities and professionals altogether rather than separately to gain a more in-depth knowledge and understanding of the facilitators and barriers in achieving reduction in aggressive challenging behaviour. In the future, studies could also attempt to identify the most important personal attributes of professionals and obtain a better understanding of the nature of the relationship between the adult with intellectual disabilities and the professional involved in their care. Finally, while psychoeducation of carers to better understand the presence of aggressive challenging behaviour is important, it is equally essential for professionals to establish positive relationships with family/paid carers and/or adults with intellectual disability, where possible, and discuss with sensitivity the degree of expected and desired change of these behaviours to increase motivation and engagement with services.

### Strengths and limitations

This study examines the experiences of multiple stakeholders about the perceived facilitators and barriers of delivering psychosocial interventions to address aggressive challenging behaviour in adults with intellectual disabilities across UK services. This is particularly important as current guidelines emphasise the use of non-pharmacological interventions for the management of behaviour that challenges and reducing the inappropriate prescription of psychotropic medications in adults with intellectual disabilities ( Branford *et al.*, 2018; NICE, 2015). This study collected a large amount of data from participants who had received individual psychosocial therapies such as CBT to network wide support including PBS to address aggressive challenging behaviour. Nonetheless, the study findings do not represent the views of adults with severe intellectual disabilities who may have a very different experience of care, because almost all suitable psychosocial interventions are provided *via* proxy. In addition, the voices of adults with mild/moderate intellectual disability remain a minority compared to carers and professionals. Collecting qualitative data from a large number of stakeholders meant that analysis of the different perspectives across the triads was also limited. Recruitment in the study began in November 2020 during the COVID-19 pandemic which might have introduced selection bias. It is likely that participants with strong views on the topic or positive relationship with professionals were keen to participate in the study. There was, also, limited representation of triads from Northern Ireland and Scotland, with only one and two triads from each country, respectively. Yet, views appear to converge across participants from all three countries and the care systems for people with intellectual disabilities are broadly organised in a similar way in the three countries. Finally, we acknowledge that this may be a limited outcome as often the emphasis is also on improving quality of life which may lead to the eventual reduction of aggressive challenging behaviour. However, the latter is the commonest reason for referral to the community intellectual disability services and many behavioural interventions are tailored to confer immediate alleviation of the risk of injury to self and/or others and to avoid other consequences such as destruction of the environment.

## Conclusion

Adults with intellectual disabilities who display aggressive challenging behaviour face numerous challenges to accessing psychosocial therapies. There is a gap in care plans being developed with input from family and/or paid carers and adults with intellectual disabilities, and the implementation of care plans in daily practice. The present study gave an overview of multiple stakeholder’s experiences of the value of therapeutic and supportive relationships and personalised care in psychosocial interventions to address aggressive challenging behaviour in adults with intellectual disabilities. It also described ongoing challenges within community intellectual disability services with negative outcomes for the implementation of psychosocial interventions. Social care funding should be at the forefront of any mental health policy briefing, and this includes establishing a good network of support to carers of adults with intellectual disabilities as well as high quality and consistent support to people with intellectual disabilities. Providing this broader input is an important and often overlooked avenue of professional support and is key to improving outcomes for those who display aggressive challenging behaviour. Nonetheless, these service improvements require shared knowledge and understanding of the need to adapt interventions for adults with intellectual disabilities among NHS staff members. Regardless of the improvements in the health and social care provision for adults with intellectual disabilities over the years, there are still serious implementation barriers that likely compromise the effectiveness of psychosocial interventions. Future research should focus on the processes and factors that facilitate high-quality and consistent support to adults with intellectual disabilities and their carers.

## Data Availability

Zenodo: Stakeholder views on the barriers and facilitators of psychosocial interventions to address reduction in aggressive challenging behaviour in adults with intellectual disabilities. https://doi.org/10.5281/zenodo.8032593 ( Kouroupa *et al.,* 2023). The project contains the following underlying data: Interview Transcripts.zip Zenodo: Stakeholder views on the barriers and facilitators of psychosocial interventions to address reduction in aggressive challenging behaviour in adults with intellectual disabilities. https://doi.org/10.5281/zenodo.8032593 ( Kouroupa *et al.,* 2023). The project contains the following extended data: Study_Topic_Guides.zip Data are available under the terms of the Creative Commons Attribution 4.0 International license (CC-BY 4.0).
