# Supplementary material for: Stakeholder views on the barriers and facilitators of psychosocial interventions to address reduction in aggressive challenging behaviour in adults with intellectual disabilities
Source: NIHR Open Res. 2023 Dec 29;3:40. Originally published 2023 Aug 14. [Version 2] doi: 10.3310/nihropenres.13437.2 (PMC10593323; doi:10.3310/nihropenres.13437.2)
Supplement: Peer review history

# Reviewer report: Reviewer response for version 1

**DOI:** 10.3310/nihropenres.14575.r30466

**Published:** 2023-10-18

**Authors:** Karl Nunkoosing, Mark Haydon-Laurelut

**Affiliations:** 1 Department of Psychology, University of Portsmouth, Portsmouth, England, UK

**Reviewer: Karl Nunkoosing, Principal Lecturer, Dept. of Psychology, University of Portsmouth.**

**Title: Stakeholder views on the barriers and facilitators of psychosocial interventions to address reduction in aggressive challenging behaviour in adults with intellectual disabilities**

 The aggressive challenging behaviours by some persons with intellectual disabilities make life difficult for the learning disabled men and women and their carers and partners. This phenomena also occupies considerable time, effort and concerns of care and therapeutic professionals. It is good to read about a research that is inclusive of all 3 stake holders. The interviews with members of the triad which includes the person with LD and CB, their carer and professional therapist or physician is a worthy extension of the study by Ramsden *et al* (2016) ^1^. This inclusive design stands out as a worthy example of research that merits wider application in intellectual disability research generally and in evaluation research particularly. This work is an example of ‘doing research with people’ rather than ‘doing research on people’.

 There are two types of readers for this research, psychosocial clinicians and researchers. The former will find much that constitute evidence about how to ensure that their interventions (not exclusively about aggressive challenging behaviours) lead to successful outcomes as well as practices that are barriers to enhancing the quality of life of intellectually disabled persons. However researchers are likely to have questions that require additional attention.

 Whilst I understand that a report such as this cannot attend to everything, attention to the following would add to the validity and replicability of this important work:

1. More information about how and where the interviews were conducted. It appears that the participants were interviewed individually. The discussion could take up the issue about the potential worth of interviewing the members of the triad together. Inevitably there are issues of research ethics that will influence such design decision.
2. The topics of the semi-structured interviews that were obtained from the ‘expert by experience’ as well as the researchers from their review of research examined in the introduction of the report.
3. Whilst the method described here is in keeping with the deductive method of Framework Thematic Analysis, this does raise issues that need attention in the discussion or limitation of the study. What were the criteria for selecting the 5 transcripts that provided the initial coding of the data? How did these 5 transcripts represent the 3 types of stake holders? I am of the view that this procedure merits further justification. When one looks for certain code in such a rich set of qualitative data, one inevitably finds what one is looking for at the risk of missing other information that may be pertinent to one’s research question/s.

The interpretations of the themes are richly supported by excerpts from the interview transcripts, however when I read the excerpts from the professionals, I am intrigued to find out about their professions…

 This research makes a significant contribution to knowledge about psychosocial interventions in the lives of intellectually disabled people, their carers/supporters and therapists. Its ethos of inclusivity and attention to the voices of intellectually disabled people, who are also ‘experts by experience’, is exemplary. Dagnan (2007) ^2^ literature review identifies psychosocial interventions as occurring at four levels. It strikes me that the discussion about ‘facilitators and barriers’ of such interventions, identified here, would benefit from addressing these levels. In particular it seems that some of the barriers are occurring at the social and service/organisational levels, whilst the facilitators occur at the individual or relational level.

 Finally, good research evokes new research questions. Questions such as:

- What are the personal attributes of talented psychosocial therapists?
- What is the nature of the relationship between the person who experiences learning difficulties and the therapist who facilitates positive outcomes for the latter?

**Additional Review from Dr Mark Haydon-Laurelut, Senior Lecturer, Department of Psychology, University of Portsmouth**

 This article explores an under researched and important area. I have a few suggestions.

 A limitation to the article is the notion of ‘psychosocial interventions. The approaches described have very significant differences ranging from individual psychological therapies such as Cognitive Behaviour Therapy (CBT) to network wide behavioural assessment and planning (Positive Behaviour Support). 

 Please consider the rewording the following: *‘Psychosocial interventions aim to meet unmet needs of adults with intellectual disabilities including housing, self-care, social and interpersonal skills, daytime activities, and housekeeping for those in family, supported or residential care homes (Gustafsson et al., 2009)’. *Therapies (for example psychological therapies such as CBT) do not routinely attempt to meet the ‘housing needs’ and ‘housekeeping’ of clients.

 Additionally, discussion of for example *:* ‘ *a gap in care plans being developed with input from family and/or paid carers and adults with intellectual disabilities, and the implementation of care plans in daily practice’ *fits clearly with some service/network approaches such as PBS and less to individual psychological therapies/psychotherapies.

 Overall the very broad categorisation of psychosocial interventions works as a limitation of the research and it is suggested this could be added to the appropriate section.

 The introduction notes that:

*‘In addition, there is scarce understanding of how those who receive these interventions experience them and what their expectations are…’ *Any studies that do exist that address this issue should be cited here.

**Methods**

 Further information on framework analysis would be useful as well as the advantages and disadvantages of mixed inductive and deductive analytic aspects.

Is the work clearly and accurately presented and does it cite the current literature?

Yes

If applicable, is the statistical analysis and its interpretation appropriate?

Not applicable

Are all the source data underlying the results available to ensure full reproducibility?

Yes

Is the study design appropriate and is the work technically sound?

Yes

Are the conclusions drawn adequately supported by the results?

Yes

Are sufficient details of methods and analysis provided to allow replication by others?

Partly

Reviewer Expertise:

Intellectual Disabilities and Qualitative Methodologies

We confirm that we have read this submission and believe that we have an appropriate level of expertise to confirm that it is of an acceptable scientific standard.

## References

[1] : Perceived barriers and facilitators to positive therapeutic change for people with intellectual disabilities: Client, carer and clinical psychologist perspectives. *J Intellect Disabil* .2016;20(3) : 10.1177/1744629515612627 241-62 10.1177/1744629515612627 26483080

[2] : Psychosocial intervention for people with learning disabilities. *Advances in Mental Health and Learning Disabilities* .2007;1(2) : 10.1108/17530180200700013 3-7 10.1108/17530180200700013

# Response

**Published:** 2023-12-02

**Authors:** Athanasia Kouroupa

**Affiliations:** Division of Psychiatry, University College London, London, England, UK

Whilst I understand that a report such as this cannot attend to everything, attention to the following would add to the validity and replicability of this important work:

 1. More information about how and where the interviews were conducted. It appears that the participants were interviewed individually. The discussion could take up the issue about the potential worth of interviewing the members of the triad together. Inevitably there are issues of research ethics that will influence such design decision.

**Our response:** Thank you for the comment. The researcher interviewed each participant separately. The study procedures section has been updated accordingly (line 125). All interviews were conducted online as a result of the COVID-19 lockdown restrictions (line 135). The discussion has been amended (line 539-545).

 2. The topics of the semi-structured interviews that were obtained from the ‘expert by experience’ as well as the researchers from their review of research examined in the introduction of the report.

**Our response:** This is an accurate reflection of the Patient and Public Involvement section.                                                  

 3. Whilst the method described here is in keeping with the deductive method of Framework Thematic Analysis, this does raise issues that need attention in the discussion or limitation of the study. What were the criteria for selecting the 5 transcripts that provided the initial coding of the data? How did these 5 transcripts represent the 3 types of stake holders? I am of the view that this procedure merits further justification. When one looks for certain code in such a rich set of qualitative data, one inevitably finds what one is looking for at the risk of missing other information that may be pertinent to one’s research question/s.

**Our response:** Thank you for your comment. The data coding and analysis section has been revised (line 154-156).

 The interpretations of the themes are richly supported by excerpts from the interview transcripts, however when I read the excerpts from the professionals, I am intrigued to find out about their professions…

**Our response:** Thank you for your comment. The quotes from professionals now include their professions.

 This research makes a significant contribution to knowledge about psychosocial interventions in the lives of intellectually disabled people, their carers/supporters and therapists. Its ethos of inclusivity and attention to the voices of intellectually disabled people, who are also ‘experts by experience’, is exemplary. Dagnan (2007) literature review identifies psychosocial interventions as occurring at four levels. It strikes me that the discussion about ‘facilitators and barriers’ of such interventions, identified here, would benefit from addressing these levels. In particular it seems that some of the barriers are occurring at the social and service/organisational levels, whilst the facilitators occur at the individual or relational level.

**Our response:** Thank you for the suggested literature. The discussion has been amended (line 506-509 and line 524).

 Finally, good research evokes new research questions. Questions such as:

1. What are the personal attributes of talented psychosocial therapists?
2. What is the nature of the relationship between the person who experiences learning difficulties and the therapist who facilitates positive outcomes for the latter?

**Our response:** Thank you for the comment. The discussion section has been updated accordingly (line 540-545).

**Additional Review** from Dr Mark Haydon-Laurelut, Senior Lecturer, Department of Psychology, University of Portsmouth

 A limitation to the article is the notion of ‘psychosocial interventions. The approaches described have very significant differences ranging from individual psychological therapies such as Cognitive Behaviour Therapy (CBT) to network wide behavioural assessment and planning (Positive Behaviour Support). 

**Our response:** Thank you for the comment. The relevant section has been amended (line 559).

 Please consider rewording the following: ‘Psychosocial interventions aim to meet unmet needs of adults with intellectual disabilities including housing, self-care, social and interpersonal skills, daytime activities, and housekeeping for those in family, supported or residential care homes (Gustafsson et al., 2009)’. Therapies (for example psychological therapies such as CBT) do not routinely attempt to meet the ‘housing needs’ and ‘housekeeping’ of clients.

 Additionally, discussion of for example: ‘a gap in care plans being developed with input from family and/or paid carers and adults with intellectual disabilities, and the implementation of care plans in daily practice’ fits clearly with some service/network approaches such as PBS and less to individual psychological therapies/psychotherapies.

**Our response:** Thank you for the comment. This is a broad definition of psychosocial interventions offered to adults with intellectual disabilities who display aggressive challenging behaviour. In this study, we do not only target individual psychosocial therapies such as Cognitive Behaviour Therapy (CBT). We also refer to network-wide behavioural support such as Positive Behaviour Support, first-line therapy in the UK NHS and other social supports that may also lead to quality of life improvements. Therefore, we have decided not to reword the sentence to be inclusive of the psychosocial support offered to this population. 

 Overall, the very broad categorisation of psychosocial interventions works as a limitation of the research, and it is suggested this could be added to the appropriate section.

**Our response:** Thank you for the comment. The relevant section has been amended (line 559).

 The discussion notes that:

 ‘In addition, there is scarce understanding of how those who receive these interventions experience them and what their expectations are…’ Any studies that do exist that address this issue should be cited here.

**Our response:** Thank you for the comment. The discussion has been amended (line 529).

 Methods

 Further information on framework analysis would be useful as well as the advantages and disadvantages of mixed inductive and deductive analytic aspects.

**Our response:** Thank you for the comment. The relevant section has been amended (lines 143-146).

# Reviewer report: Reviewer response for version 1

**DOI:** 10.3310/nihropenres.14575.r30307

**Published:** 2023-08-29

**Authors:** Romina Rinaldi

**Affiliations:** 1 Department of Clinical Orthopedagogy, Universite de Mons, Mons, Walloon Region, Belgium

This study explored factors affecting the success of interventions for reducing aggressive behavior in adults with intellectual disabilities. It found that strong therapeutic relationships and personalized care facilitate positive change, while differences in expectations and structural issues hinder it. To improve outcomes, operational changes like refining referral criteria and addressing workforce challenges are recommended. 

 The scientific literature is properly integrated and cited. However, it would be useful to better define what is meant by "psychosocial intervention" within the introduction. Indeed, this term can cover relatively broad practices and services, and insofar as the question of access and that of evidence-based practices are raised later in the discussion, it would be useful to be able to better delimit the concept and its applications.

 Study design and methodological options are appropriate to the research questions. A strength of the study is the use of an inclusive research design involving panels of people with intellectual disabilities and carers in the design of the study, as well as considering the perspective of triads (people with intellectual disabilities-carer-professional).

 Overall, the manuscript provides sufficient details of methods and analysis for replication. However, some missing information would appear to be relevant.

 It wasn't entirely clear to me to what extent the stakeholders and/or the panel of experts by experience had been involved in analysing the data. Besides, I did not fully understand if participants were actually met once (in “triads”) or did the researcher met each stakeholder at different occasion.

 Providing an overview of the interview guide would be interesting for future research.

 It would be interesting to have a better idea of the relative weight of each theme and sub-theme within the analyses. In addition, the discussion mentions that certain issues were more relevant for people with intellectual disabilities than for carers or professionals, and vice versa; but the presentation of the results as they stand does not make it possible to determine in which theme or sub-themes the units of meaning linked to the discourse of people with intellectual disabilities were more or less present. This information would make it easier to understand the data.  

 Similarly, if relevant, it would be useful to have a perspective in relation to 'sites', as it is suggested that perspectives are consistent across these in the discussion.

Is the work clearly and accurately presented and does it cite the current literature?

Yes

If applicable, is the statistical analysis and its interpretation appropriate?

Not applicable

Are all the source data underlying the results available to ensure full reproducibility?

Yes

Is the study design appropriate and is the work technically sound?

Yes

Are the conclusions drawn adequately supported by the results?

Yes

Are sufficient details of methods and analysis provided to allow replication by others?

Partly

Reviewer Expertise:

Disability studies

I confirm that I have read this submission and believe that I have an appropriate level of expertise to confirm that it is of an acceptable scientific standard.

# Response

**Published:** 2023-12-02

**Authors:** Athanasia Kouroupa

**Affiliations:** Division of Psychiatry, University College London, London, England, UK

The scientific literature is properly integrated and cited. However, it would be useful to better define what is meant by "psychosocial intervention" within the introduction. Indeed, this term can cover relatively broad practices and services, and insofar as the question of access and that of evidence-based practices are raised later in the discussion, it would be useful to be able to better delimit the concept and its applications.

**Our response:** Thank you for the comment. The manuscript provides a definition in line 20-22.

 It wasn't entirely clear to me to what extent the stakeholders and/or the panel of experts by experience had been involved in analysing the data. Besides, I did not fully understand if participants were actually met once (in “triads”) or did the researcher met each stakeholder at different occasion.

**Our response:** Thank you for the comment. The advisory groups were actively involved at all stages of the study (line 85-90). They also reviewed and commented on the emerging themes and sub-themes during the coding phase. The researcher met each participant separately to conduct 1:1 interviews. The procedures section has been updated accordingly (line 125).

 Providing an overview of the interview guide would be interesting for future research.

**Our response:** Thank you for your comment. The topic guides are available via Zenodo (see data availability section).

 It would be interesting to have a better idea of the relative weight of each theme and sub-theme within the analyses. In addition, the discussion mentions that certain issues were more relevant for people with intellectual disabilities than for carers or professionals, and vice versa; but the presentation of the results as they stand does not make it possible to determine in which theme or sub-themes the units of meaning linked to the discourse of people with intellectual disabilities were more or less present. This information would make it easier to understand the data.  

 Similarly, if relevant, it would be useful to have a perspective in relation to 'sites', as it is suggested that perspectives are consistent across these in the discussion.

**Our response:** Thank you for your comment. In this study, our aim was to provide a range of views and perspectives from multiple stakeholder members in intellectual disabilities. In keeping with other qualitative research, we have decided not to incorporate numbers (or weights) as it can lead to inferences about the generality of findings, which may be inaccurate or not justified given the interpretative nature of the analysis.
